# Oral nicotinamide mononucleotide (NMN) to treat chronic insomnia: protocol for the multicenter, randomized, double-blinded, placebo-controlled trial

**DOI:** 10.1186/s13063-023-07351-8

**Published:** 2023-05-18

**Authors:** Xiangyang Gao, Junhua Li, Sanping Xu, Xueying Li, Xicheng Wang, Yongli Li, Yan Huang, Shaohui Liu, Qiang Zeng

**Affiliations:** 1 Health Management Institute, The Second Medical Center & National Clinical Research Center for Geriatric Diseases, Chinese PLA General Hospital, Beijing, China; 2 Health Management Center, Handan Central Hospital, Hebei Province, Handan, China; 3 Health Management Center, Union Hospital, Tongji Medical College, Huazhong University of Science and Technology, Hebei Province, Wuhan, China; 4 Department of Biostatistics, Peking University First Hospital, Beijing, 100034 China; 5 Beijing Dublin International Collage, Beijing University of Technology, Beijing, 100124 China; 6 Health Management Center, Henan Provincial People’s Hospital, Zhengzhou, China; 7 Health Management Center, West China Hospital, Sichuan University, Sichuan Province, Chengdu, China; 8 Health Management Center, Xiangya Hospital, Central South Hospital, Hunan Province, Changsha, China

**Keywords:** Chronic insomnia, Nicotinamide mononucleotide (NMN), Randomized controlled trial

## Abstract

**Background:**

The treatment of insomnia, which is the most common sleep disorder, includes drug and behavioral treatment, but each treatment measure has its limitations. So new treatment method needs to be taken to improve the treatment effect. MN supplementation is a potential promising new method for the treatment of insomnia, resulting in a rising need for methodological research towards verifying its efficacy.

**Methods/design:**

We describe a proposal for a multicenter, patient-assessor-blinded, randomized controlled trial with two parallel arms. A total of 400 chronic insomnia patients will be allocated 1:1 to the intervention group (treatment with oral NMN 320 mg/day) or control group (treatment with oral placebo). All subjects are clinical chronic insomnia patients who meet all inclusion criteria. All subjects are treated by taking NMN or placebo.

The primary outcome is the score on the Pittsburgh Sleep Quality Index (PSQI). Secondary outcomes are the score on the Insomnia Severity Index (ISI) and Epworth Sleeping Scale (ESS), the total sleep time (TST), sleep efficiency (SE), sleep latency, and REM sleep latency to assess sleep quality changes.

Subjects are assessed at two time points: baseline and follow-up. The duration of the clinical trial is 60 days.

**Discussion:**

This study will provide more evidence on the effects of NMN on improving sleep quality among patients with chronic insomnia. If proven effective, NMN supplement can be used as a new treatment for chronic insomnia in the future.

**Trial registration:**

Chinese Clinical Trial Registry (chictr.org.cn) ChiCTR2200058001. Registered on 26 March 2022.

## Administrative information

The numbers in curly brackets in this protocol refer to the Standard Protocol Items: Recommendations for Interventional Trials (SPIRIT) checklist item numbers. The order of the items has been modified to group similar items (see http://www.equator-network.org/ reporting-guidelines/spirit-2013-statement-defining- standard-protocol-items-for-clinical-trials/) (Tables 1 and 2).

**Table 1:** Administration information

Title {1}	Oral nicotinamide mononucleotide (NMN) as a treatment for chronic insomnia: protocol for the multicenter, randomized, double-blind, placebo-controlled trial
Trial registration {2a and 2b}	Chinese Clinical Trial Registry (chictr.org.cn): ChiCTR2200058001. Registered on 26 March 2022. ChiCTR2200058001
Protocol version {3}	13 March 2022. Version 1
Funding {4}	This trial is funded by grants from China Health Promotion Foundation
Author details {5a}	Xiangyang Gao, Health Management Institute, The Second Medical Center & National Clinical Research Center for Geriatric Diseases, Chinese PLA General Hospital, Beijing, China Junhua Li, Health Management Center, Handan Central Hospital, Handan, Hebei Province, China Sanping Xu, Health Management Center, Union Hospital, Tongji Medical College, Huazhong University of Science and Technology, Wuhan, Hubei Province China Xueying Li, Department of Biostatistics, Peking University First Hospital, Beijing 100,034, China Xicheng Wang, Beijing Dublin International Collage, Beijing University of Technology, Beijing 100,124, China Yongli Li, Health Management Center, Henan Provincial People's Hospital, Zhengzhou, China Yan Huang, Health Management Center, West China Hospital, Sichuan University, Chengdu, Sichuan Province, China Shaohui Liu, Health Management Center, Xiangya Hospital, Central South Hospital, Changsha, Hunan Province, China Qiang Zeng, Health Management Institute, The Second Medical Center & National Clinical Research Center for Geriatric Diseases, Chinese PLA General Hospital, Beijing, China XYG and ZQ are the Chief Investigators who conceived the study, led the proposal and protocol development. JHL, SPX, XYL, XCW, YLL, SHL, and HY all contributed to the trial design and to development of the proposal. XYL and XCW are lead trial methodologist. All authors read and approved the final protocal manuscript
Name and contact information for the trial sponsor {5b}	This trial is funded and proceeded by China Health Promotion Foundation Wu Shiyong, Room 521,Wankai Road, Fentai District, Beijing, China, + 86–13,261,008,988, Email: 82,125,586@qq.com
Role of sponsor {5c}	This trial is solely funded by non-commercial sources. Funding sources have had no role in the design of this study and will not have any role during its execution, analyses, interpretation of the data, or decision to submit results

**Table 2:** Registration data

Data category	Information
Primary registry and trial identifying number	ChiCTR2200058001
Date of registration in primary registry	March 26, 2022
Secondary identifying numbers	NA
Source(s) of monetary or material support	China Health Promotion Foundation
Primary sponsor	China Health Promotion Foundation
Secondary sponsor(s)	NA
Contact for public queries	Wu Shiyong (Email: 82,125,586@qq.com)
Contact for scientific queries	Qiang Zeng, MD (Email: zq301@126.com)
Public title	Oral nicotinamide mononucleotide (NMN) as a treatment for chronic insomnia: protocol for the multicenter, randomized, double-blind, placebo-controlled trial
Scientific title	Oral nicotinamide mononucleotide (NMN) as a treatment for chronic insomnia: protocol for the multicenter, randomized, double-blind, placebo-controlled trial
Countries of recruitment	China
Health condition(s) or problem(s) studied	Chronic insomnia
Intervention(s)	Active comparator: Nicotinamide mononucleotide (320 mg/day) Placebo comparator: Placebo
Key inclusion and exclusion criteria	Inclusion criteria: Age between 18 and 65 years; clinical diagnosis of chronic insomnia (according to the ICSD-3 criteria for chronic insomnia)
	Exclusion criteria: Physical diseases, mental disorders, and/or sleep disorders; received any insomnia drugs and psychotherapy within the preceding 1 month; on a continuous current daily intake of nutritional supplements and vitamin supplements
Study type	Interventional
	Multicenter, double-blinded, randomized controlled superiority trial with a two-group parallel design
	Phase III
Date of first enrolment	April 2022
Target sample size	400
Recruitment status	Recruiting
Primary outcome(s)	Pittsburgh Sleep Quality Index (PSQI)
Key secondary outcomes	Epworth Sleeping Scale (ESS), Insomnia Severity Index (ISI), Total sleep time (TST), sleep efficiency (SE), sleep latency, REM sleep latency, the percentage of N1, N2, N3, and REM

## Introduction

### Background and rationale {6a}

#### Insomnia

Insomnia refers to persistent challenges in falling asleep, maintaining sleep, or waking up earlier than the habitual rise time, and is associated with the impairment of daytime functioning despite the opportunity for sufficient sleep duration [1]. It is a prevalent condition associated with significant morbidity, reduced productivity, increased risk of accidents, and poor quality of life [2]. The prevalence of insomnia disorder is approximately 10–20%, of which approximately 50% have a chronic course [3]. Based on a meta-analysis of China’s population, the prevalence of insomnia was 15% [4]. Of the Chinese survey respondents, 45.4% reported varying degrees of insomnia [5]. Insomnia has become the second major reason that leads patients to visit neurology clinics, the first one is headaches [6]. Owing to its prevalence, the annual loss of quality-adjusted life-years (QALYs) from insomnia appears to be greater than that from other medical and psychiatric conditions, including arthritis, depression, and hypertension [7]. As the most common sleep disorder, insomnia is a substantial burden on healthcare systems and vulnerable patient groups. The combined direct and indirect costs for insomnia in the United States exceed $100 billion annually [8].

Currently, the prescribed treatment for insomnia primarily involves drug and behavioral treatments, however, they have limitations [9]. Many patients prefer hypnotics to non-drug treatments despite side effects and issues with drug dependence and tolerance because they help reduce sleep latency (SL) and increase total sleep time (TST) at night [10, 11]. Multifactorial behavioral treatments such as cognitive behavioral therapy for insomnia (CBTI) have moderate to great effects on sleep parameters; however, only 26–43% of patients experience complete relief from insomnia [12]. Moreover, the high cost and lack of trained providers of CBTI have prevented widespread uptake [13–15]. Therefore, the public health burden of insomnia is still large, and new treatment methods must be developed to improve it.

Insomnia is a complex interaction between psychological cognitive arousal, altered circadian clock, and sleep homeostatic mechanisms. Circadian factors promote wakefulness on a roughly 24-h biological clock, whereas homeostatic factors respond to accumulated wakefulness with the drive for sleep [16, 17]. Disrupted sleep–wake and molecular circadian rhythms are features of aging associated with metabolic diseases and reduced nicotinamide adenine dinucleotide (NAD +) levels [18]. A clue to the mechanism underlying the decline in circadian rhythms and metabolism with age stems from the observation that the longevity-associated deacetylase, SIRT1, interacts with the core clock complex [19, 20]. For SIRT1 activity to occur, the sentinel metabolite NAD + , which declines with age is required. Declining NAD + /SIRT1 leads to the downregulated expression of clock genes, such as BMAL1 and PER2, which affects the internal circadian clock [21–23]. Neural activation dependent on SIRT1 in the dorsomedial hypothalamic nucleus and lateral hypothalamus has been reported to prevent aging-induced decline in the quality of sleep-in mice [24]. Nicotinamide mononucleotide (NMN) is an intermediate that effectively improves the level of NAD + [22] and has been extensively studied over the years [25]. Therefore, NMN supplementation is a promising method to treat insomnia. Recent preclinical studies have demonstrated that administering NMN could compensate for NAD + deficiency and can affect diverse pharmacological activities in various diseases, including diabetes, obesity, ischemia reperfusion injury (IRI), and heart failure [26–29]. Given that NMN has demonstrated high efficacy and benefits in various mouse models of human disease, several clinical trials have been conducted to investigate its clinical applicability [30, 31]. Therefore, it is necessary to verify the of NMN’s effect on insomnia. This trial aims to prove that NMN supplementation is an effective treatment for therapy chronic insomnia, a subtype of insomnia, and refers to symptoms occurring 3 or more times per week for 3 months or longer [16].

### Objectives {7}

Our primary objective is to evaluate the efficacy and safety of nicotinamide mononucleotide (NMN) as a treatment for chronic insomnia.

### Trial design {8}

**Fig. 1:**
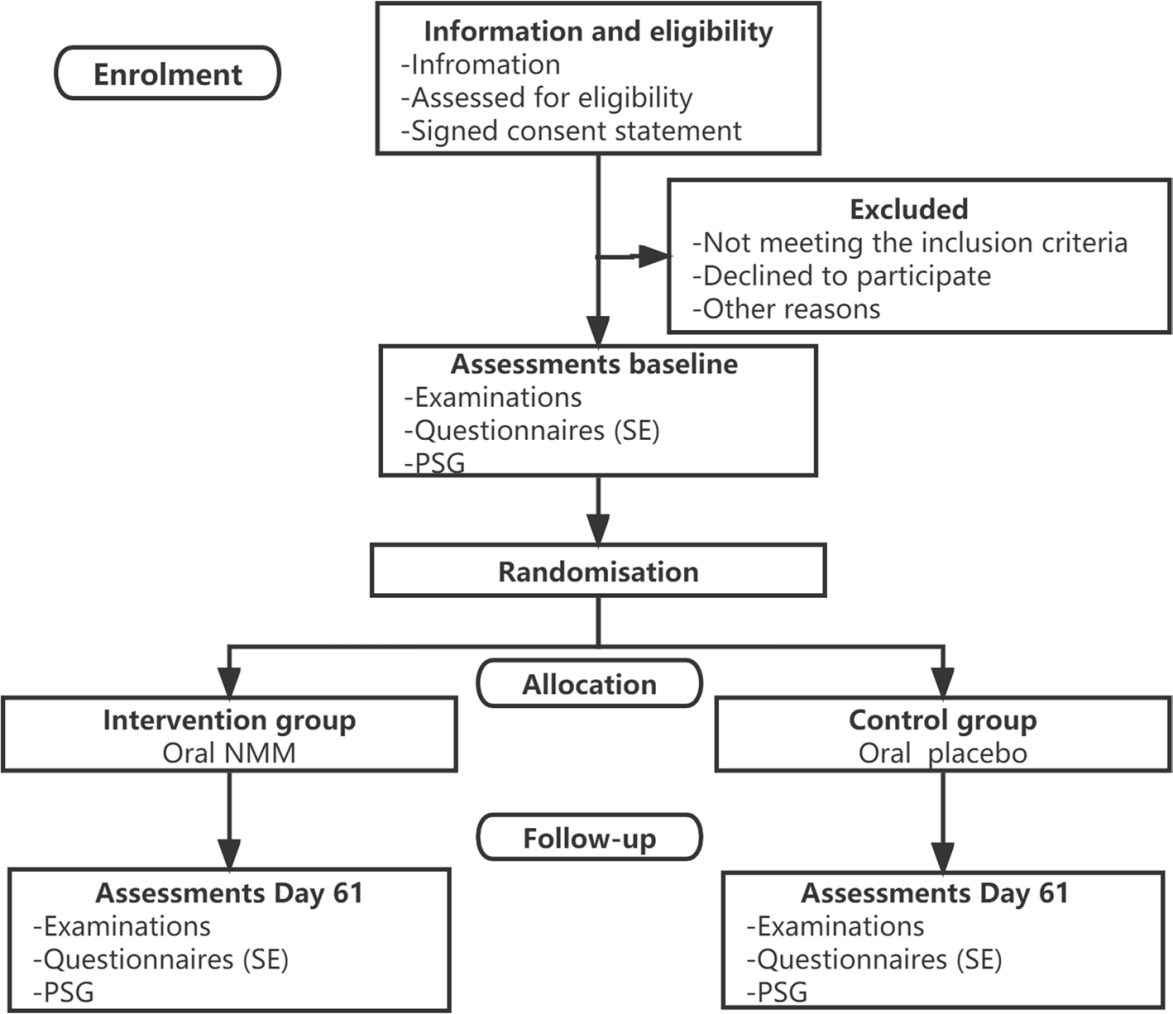
Study flowchart

This trial will be carried out as a multicenter, double-blinded, randomized controlled superiority trial with a two-group parallel design. Participants (*n* = 400) will be allocated 1:1 to either oral MNM as treatment (intervention group) or to oral placebo (control group). Outcome assessments will be conducted two times for each participant: at baseline and at follow-up (day 60), as presented in Fig. 1.

## Methods: participants, interventions, and outcomes

### Study setting {9}

We will recruit patients from the below nine hospitals:

1. Health Management Center, Union Hospital, Tongji Medical College, Huazhong University of Science and Technology
2. Health Management Center, Handan Central Hospital
3. Health Management Center, The Second Affiliated Hospital of Xi’an Jiaotong University
4. Health Management Center, Liaoning Electric Power Central Hospital
5. Health Management Center, The Fourth People’s Hospital of Zigong City
6. Health Management Center, Guang’an People's Hospital
7. Health Management Center, The Second People’s Hospital of Weinan City
8. Health Management Center, Three Gorges Hospital affiliated to Chongqing University
9. Health Management Center, Hunan Provincial People’s Hospital

### Eligibility criteria {10}

#### Inclusion criteria

Participants will be included if they:

1. Age between 18 and 65 years.
2. Clinical diagnosis of chronic insomnia (according to the ICSD-3 criteria for chronic insomnia).
3. Without hepatorenal and cardiac dysfunction (normal laboratory examination, ECG, etc.)
4. Can be trained and able to use the smart wearable device.
5. Be able to read, understand, and provide written informed consent before enrolling in the study.
6. Agree to participate for the entire study period.

#### Exclusion criteria

Patients will be excluded if they:

1. Physical diseases, mental disorders, and/or sleep disorders (including patients with restless legs syndrome) that are clearly diagnosed, directly affect sleep, and are not cured.
2. Received any insomnia drugs and psychotherapy within the preceding 1 month.
3. On a continuous current daily intake of nutritional supplements and vitamin supplements.
4. Pregnant, shift workers, perennial night shift workers, frequent cross-timezone pilots (such as crew members of international flights), and patients with sleep phase delay syndrome.
5. Cancer, dyslexia, or lack of self-care ability.
6. Depression and suicide risk (suicide plan/attempt within the preceding 1 month).
7. Regular medicated background systemic chronic diseases, such as hypertension and diabetes.
8. Hyperuricemia or gout.
9. Sleep disorders caused by other diseases.

### Who will take informed consent? {26a}

The fully trained specific researchers who are on-site will obtain informed consent from participants.

### Additional consent provisions for collection and use of participant data and biological specimens {26b}

On the consent form, participants are asked to sign if they agree to have been given written and oral information on the purpose, method, advantages, and disadvantages of saying yes to participate in the trial, and if they agree to participate voluntarily. On the consent form, participants are informed that they can withdraw the consent without losing any rights to treatment, either current or future. They are also asked if they agree to the use of their data when they choose to withdraw from the trial.

This trial does not involve collecting biological specimens for storage.

## Interventions

### Explanation for the choice of comparators {6b}

Participants in the control group will take the oral placebo which is manufactured where same as the intervention oral NMN.

Chronic insomnia is not a life-threatening disease, instead, patient’s emotional and mental status can be contributed. The prescribed treatment for chronic insomnia primarily involves drug and behavioral treatments both have limitations, thus, to use placebo as the control group.

### Intervention description {11a}

In this trial, the intervention group (*n* = 200) will have oral MNM (320 mg/day), 1 tablet bid(each tablet contains 160 mg NMN), after the meal for 60 days. The control group (*n* = 200) will have oral placebo, 1 tablet bid, after the meal for 60 days.

### Criteria for discontinuing or modifying allocated interventions {11b}

Interventions could be discontinued or modified under the below circumstances:

- Allergic reactions: If allergic skin reactions have been observed, discontinue allocated interventions. Meanwhile, this should be reported as an adverse event.
- Headache: If headache has been observed, temporary suspension of the allocated intervention until the symptom disappears is recommended. If the headache re-happens, discontinue allocated interventions. Meanwhile, this should be reported as an adverse event.
- Nausea: If nausea has been observed, temporary suspension of the allocated intervention until the symptom disappears is recommended. If the headache re-happens, discontinue allocated interventions. Meanwhile, this should be reported as an adverse event.

### Strategies to improve adherence to interventions {11c}

Adherence enhancement strategies are in use throughout the trial period, e.g., patients are being reminder of the importance of adhering regardless of allocation via phone call on day 1, day 3, day 7, day 15, day 30, day 45, and day 60.

### Relevant concomitant care permitted or prohibited during the trial {11d}

Throughout the trial period, participants regardless of allocation, who had background disease are permitted to have relevant medicines accordingly but are prohibited from taking together with the intervention. Participants regardless of allocation are prohibited from taking any treatment may impact the objective function e.g., anti-oxide, sleep improvement, visual fatigue reliving, body fatigue relieving).

### Provisions for post-trial care {30}

The trial sponsor will provide free medical treatment or make compensation/compensation according to relevant Chinese laws to fulfill the needs related to direct consequence of trial participation (e.g., intervention-related harms). The sponsor has insurance to cover for harms associated with the interventions. The insurance will cover for additional health care, compensation, or damages either provided voluntarily by the Sponsor, or by claims pursued through the standard process.

### Outcomes {12}

#### Primary outcome

The primary outcome is the mean change of score between baseline and follow-up after 60 days of treatment which indicated sleep efficiency. It will be assessed with the Pittsburgh Sleep Quality Index (PSQI) questionnaire. It includes nine items/18 questions with seven categories, including subjective sleep quality, sleep latency, sleep duration, habitual SE, sleep disturbances, use of sleep medications, and daytime dysfunction. A global sum score for the PSQI > 5 indicates poor sleep. The Cronbach’s α value for the PSQI is 0.83 [32, 33].

#### Secondary outcome

Other sleep efficiency indices, including the Epworth Sleeping Scale (ESS) and Insomnia Severity Index (ISI), the mean change of score will be used at the baseline and follow-up after 60 days of treatment for the secondary outcome assessment. The ESS consists of 8 self-rated items, each scored from 0–3, that measure a subject's habitual “likelihood of dozing or falling asleep” in common situations of daily living. No specific time frame is specified. The ESS score represents the sum of individual items and ranges from 0 to 24. Values > 10 are considered to indicate significant sleepiness. The ESS is sensitive to change in clinical status, as evidenced by improvements following treatment of sleep apnea with continuous positive airway pressure (CPAP). Ten psychometric analyses of the ESS support its internal consistency. The ISI is a 7-item self-report questionnaire that assesses the nature, severity, and impact of insomnia. The dimensions are severity of sleep onset, sleep maintenance and early morning waking problems, sleep dis- satisfaction, interference of sleep difficulties with daytime functioning, noticeability of sleep problems by others, and distress caused by sleep difficulties. It is rated on a 5-point Likert scale (total score 0–28). A score of 0–7 indicates absence of insomnia, 8–14 sub-threshold insomnia, 15–21 moderate insomnia, and 22–28 severe insomnia. Internal consistency is excellent in community as well as clinical samples (Cronbach’s alpha 0.90–0.91 [33, 34]).

Secondary outcomes also include the mean change from the baseline to follow-up after 60 days of treatment in total sleep time (TST), sleep efficiency (SE), sleep latency, REM sleep latency, the percentage of N1, N2, N3, and REM which been assessed by polysomnography (PSG, Model: NS-100A). PSG is a comprehensive study of the biophysiological changes that occur in the human body during sleep. The electrodes will be attached to each participant, recording data from electroencephalogram, eye movements electrooculogram, electromyogram muscle activity or skeletal muscle activation, heart rhythm, respiratory airflow, respiratory effort, and peripheral pulse oximetry. All PSG studies will be conducted in the participants’ own homes. Data from PSG will be analyzed by the researcher.

#### Safety outcome

The primary safety outcome s the incidence of adverse events. Physical examinations, laboratory tests and abdominal ultrasonography will be performed on baseline and after 60 days of treatment. The physical examination includes blood pressure, height, weight, and heart rate. Laboratory tests include urine routines, blood routines, liver and renal function, and ECG. Adverse events (AE) will be recorded in CRF; “adverse event record” of CRF includes adverse event occurrence time, end time, duration, severity, measurement taken, and outcome, and made judgment on the relationship between adverse events and interventions.

### Participant timeline {13}

The trial period for each participant is approximately 60 days. All participants will complete the same outcome assessments at baseline and at follow-up (day 61). All participants will complete the same outcome assessments at baseline, as presented in Fig. 1 and Table 3.

**Table 3:**
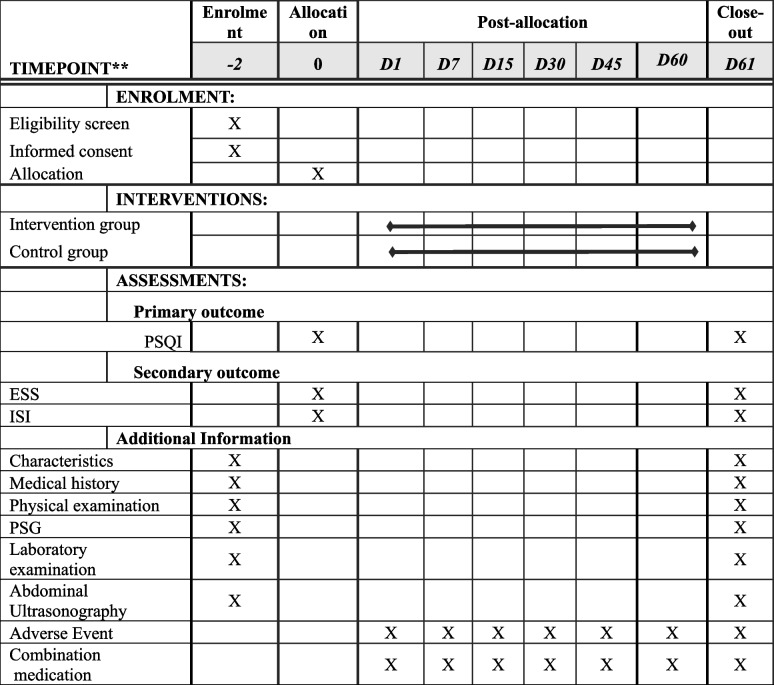
Standard protocol items

### Sample size {14}

This trial is designed as a placebo-controlled, parallel-group, and superiority trial.

Test hypothesis: H0: μT-μC ≤ 0; H1: μT-μC > 0, μT is the mean value of the intervention group’s PSQI score; μC is the mean value of control group’s PSQI score;

The ratio between groups was 1:1; α = 0.025 (one-sided), β = 0.20. According to the a previous study [35], the mean of PSQI score was 10.42, and standard deviation 3.214 in the insomnia patients. A conservative estimate based on pretest study indicated that the decrease of PSQI score in the treatment group is 1 point higher than that in the control group, and the common standard deviation is about 3.3 points. Thus, by PASS 16.0, 172 effective cases need to be completed in each group. The sample size estimation formula used by PASS is the following:

$$n=\frac{2{\left({Z}_{1-\mathrm{\alpha }}+{Z}_{1-\beta }\right)}^{2}{\sigma }^{2}}{{D}^{2}}$$

where n is the sample size of each comparison group,$\sigma$ is the common standard deviation; D is the estimated value of the difference between the average values of the two groups. Considering the possible dropout, a total of 400 cases are planned to be enrolled. There were 200 cases in each group.

### Recruitment {15}

Participants will be recruited through advertisement and referrals. Ads will be put in social media (e.g., WeChat) and poster and flyers in public places (e.g., health management center, out-patient department). Pharmacists and general practitioners around the trial sites will be contacted for potential referrals.

## Assignment of interventions: allocation

### Sequence generation {16a}

Randomization will be conducted after baseline assessment by computer-generated random numbers.

### Concealment mechanism {16b}

Sealed envelopes according to the random table are prepared. Subjects who met the inclusion criteria opened the corresponding sealed envelopes in the order of enrollment and were assigned to the study group according to the assignment on the envelopes.

### Implementation {16c}

An external collaborator (statistician) will generate the allocation sequence. This collaborator is not involved in outcome assessments or the intervention. On-site researchers will be responsible for the enrollment and assignment.

### Assignment of interventions: blinding who will be blinded {17a}

An external collaborator (statistician) will generate the blind codes for intervention and placebo which are identical. Both the investigator and the participants will be blinded at recruitment and during the intervention. The outcome assessor and data analyst will be blinded as well. Trial participants and care providers will be blinded during the study. The sequence will be stored in a password-protected data management system and cannot be accessed by blinded study staff who have contact with participants.

### Procedure for unblinding if needed {17b}

In the event of a serious adverse event (SAE) or reaction, the allocation list will be retrieved to reveal the participant’s allocated treatment during the trial, PI and on-site researchers will be responsible for signature and documentation. The process needs to be reported to Ethics Committee.

## Data collection and management

### Plans for assessment and collection of outcomes {18a}

Outcome assessors will be fully trained in the study, who pass the assessment for assessor can be responsible for outcome assessment. All the involved sites will be calibrated on laboratory test.

Each center’s assessors will be fully trained in the study requirements, standardized measurement of weight, height, blood pressure, and ECG, requirements for laboratory specimen collection including blood and urine samples, counseling for questionnaires, and the eliciting of information from study participants in a uniform reproducible manner. The blood and urine samples will be exanimated in the laboratories of each center and all the laboratories will be calibrated by the standardized procedure. All questionnaires used in the study are validated and consolidated in the literature.

All the questionnaire data, PSG data, and laboratory examination data will be collected on paper case report forms (CRFs) by assessors in each center. And range and consistency checks will be conducted on the data promptly by the research team.

These data are required to be uploaded into EDC system within one week after collecting. Coordinators in each center will be trained to be able to use the EDC system. Entering data forms, responding to data discrepancy queries and general information about obtaining research quality data will also be covered during the training session.

Data collection forms can be found in the CRFs of this trial. Email to the contact point for scientific queries can be an approach to have the above forms.

### Plans to promote participant retention and complete follow-up {18b}

Dates for assessments will be planned by on-site researchers together with each participant, and all participants will be able to connect with the responsible researcher on their telephone or an email. Participants are aware of being able to get allowance when completing all rescheduled follow-ups.

### Data management {19}

All participants will receive an identification code and all data will be de-identified. A list of identifiable participant information associated with each identification code will be stored electronically separately from the research data for 5 years. Patient-reported information will be completed electronically on a tablet, which will be used only for participants in this trial. A special Electric Data Capture (EDC) will be established for data management.

### Confidentiality {27}

All information collected during the trial will be kept confidential. All data will be processed confidentially, personal sensitive data (full name, contact information, ID) will not be included in the EDC system.

### Plans for collection, laboratory evaluation, and storage of biological specimens for genetic or molecular analysis in this trial/future use {33}

There will be no biological specimens collected for genetic or molecular analysis in this trial.

## Statistical methods

### Statistical methods for primary and secondary outcomes {20a}

Data will be evaluated by using the SPSS software V19.0 (IBM SPSS Statistics, IBM Corp, Somers, NY, USA). All demographic and baseline characteristics will be analyzed by using different approaches. The demographic information between the two groups will be compared by using a chi-square test. Differences between group means will be assessed using an independent samples *t* test (*P* < 0.05). The main objective is to assess the difference in change in PSQI score between the intervention group and control group from baseline to day 61; an analysis of the covariance model will be used for comparison in which the roles of grouping and center were also considered. The significance level was set at* P* < 0.05 and post hoc analyses were performed where appropriate. For secondary outcome data such as ISI, ESS, TST, sleep efficiency (SE), SL, and rapid eye movement (REM) sleep latency, an independent samples *t* test (*P* < 0.05) will be used to compare differences between the two groups, and a paired *t* test (*P* < 0.05) will be used to compare patients in the same group before and after treatment.

All analyses will be pre-specified in a detailed statistical analysis plan.

### Interim analyses {21b}

This trial is not subject to interim analyses.

### Methods for additional analyses (e.g., subgroup analyses) {20b}

We have no intention to conduct subgroup and adjusted analyses.

### Methods in analysis to handle protocol non-adherence and any statistical methods to handle missing data {20c}

Subjects who participated in the randomized group, whether they received treatment in that group or not, were eventually included in the assigned group for statistical analysis of efficacy, and this trial followed intention-to-treat analysis (ITT) principles[36]. Last observation carry forward (LOCF) will be used for missing data management.

### Plans to give access to the full protocol, participant-level data and statistical code {31c}

The datasets analyzed during this trial are available from the corresponding author on reasonable request.

## Oversight and monitoring

### Composition of the coordinating center and trial steering committee {5d}

A joint Trial Steering Committee (TSC) and Data Monitoring Committee (DMC) will provide supervision for the trial, providing advice to the Chief and Co-investigators on all aspects of the trial conduct and affording protection for patients by ensuring the trial is conducted according to the Medical Research Council (MRC) Guidelines for Good Clinical Practice in Clinical Trials. The TSC/DMC will be chaired by an academic clinician independent of the trial plus three other members plus the chief investigator, trial manager, statistician, and health economist.

### Composition of the data monitoring committee, its role and reporting structure {21a}

#### Refer to above {5d}

### Adverse event reporting and harms {22}

Participants will be monitored throughout the period of the intervention to detect any unintended events.

An adverse event refers to any untoward medical occurrence which happens during the trial. The study will monitor for the following NMN-related adverse events (AEs) will be monitored through routine follow-ups, including allergic reactions, headache, and nausea. Besides, other AEs will also be reported and recorded in CRF; “adverse event record” of CRF includes adverse event occurrence time, duration, end time, severity, measures taken and outcome, and make judgment on the relationship between adverse events and interventions. As safety outcome measures, AEs will be used to evaluate the safety of the intervention.

### Frequency and plans for auditing trial conduct {23}

Clinical trial auditing is the primary contact between the sponsor and investigator. In the clinical trial, the sponsor will appoint a few clinical auditors with professional medical or pharmaceutical backgrounds. The auditors must follow the standard operating procedures for clinical research; visit the research unit regularly or according to the ground reality; audit the progress and progress of clinical trials; and check and confirm that the records and reports of all the data and case report forms are correct, complete and consistent with the original data. To ensure that the clinical trial is conducted according to the clinical trial scheme, the researcher will actively cooperate with the auditors. The auditor must be responsible for the below:

1. Confirm (before the test) that the test undertaking unit has appropriate conditions including personnel allocation and training; ensure that the laboratory is fully equipped and operational; and has various inspection conditions related to the test. They must estimate if there are sufficient subjects and participating researchers are familiar with the test scheme’s requirements.
2. Monitor (during the experiment) the researcher’s implementation of the experimental scheme, confirm that the informed consent of all subjects was obtained (before the experiment), understand the enrollment rate of subjects and progress of the experiment, and confirm that the selected subjects are qualified.
3. Confirm that all data records and reports are correct and complete, and all case report forms are filled correctly and consistent with the original data. They must also ensure that all errors and omissions are corrected, noted, signed, and dated by the investigator. Dose or treatment changes, combined medication, intermittent diseases, loss of follow-up, examination omission, etc. of each subject must be confirmed and recorded. Furthermore, they must ensure that the withdrawal and loss of follow-up of the selected subjects have been explained in the case report form.
4. Confirm that all adverse events are recorded, and serious adverse events are reported and recorded within the specified time. They must verify that the intervention products for the test are supplied, stored, distributed, and recovered in accordance with relevant laws and regulations, and make corresponding records.
5. Assist the researcher in the necessary notification and application processes and report the test data and results to the sponsor.
6. Clearly and truthfully record the follow-up, test, and inspection that the researcher fails to perform, and document whether the errors and omissions were corrected.
7. Complete a written audit report (after each visit) which states the date and time of the audit, name of the supervisor, findings of the audit, etc.

### Plans for communicating important protocol amendments to relevant parties (e.g., trial participants, ethical committees) {25}

In this trial, if it is necessary, the sponsor and the main researcher can organize the amendments of the protocol. The amended protocol can take effect only after it is signed and aligned by the sponsor and the main researcher and approved by the ethics committee.

### Dissemination plans {31a}

We plan to publish one scientific paper in peer-reviewed journals based on the trial and to disseminate the results to patient organizations and the public through printed and electronic media. The outcome of this trial will be reported and presented at national and international conference.

## Discussion

This trial is designed to investigate the efficacy of oral NMN to treat chronic insomnia compared to placebo. To our knowledge, this is the first trial that has a stringent design with randomization and double-blinding to investigate evidence targeting chronic insomnia by intaking NMN supplements. The COVID-19 pandemic could pose a challenge to this trial by impacting the inclusion and follow-up of patients. To guarantee patient safety and reduce the risk of COVID-19 infection and spread, we will fully utilize digital platforms such as WeChat and telephone and limit in-person visits.

Chronic insomnia is a prevalent condition associated with significant morbidity, reduced productivity, increased risk of accidents, and poor quality of life. The current first-line treatment is burdensome to some patients. If the hypothesis is verified (i.e., NMN is relatively safe and more effective than placebo), it may be recommended to fulfill the unmet medical needs of patients with chronic insomnia.

The strengths of this trial are:

- The use of a randomization scheme with allocation concealment and double blinding.
- The use of a placebo similar in appearance and taste to the treatment.
- A comprehensive assessment for sleep efficiency.

## Trial status

This is protocol version 1, 2 Aug 2021.

Recruitment started on 1 April 2022, with an anticipated primary completion date of 31 Dec 2022.

## Data Availability

The datasets generated and/or analysed during the study are available from the corresponding author on reasonable request. The Data Management Coordinating Center will oversee the intra-study data sharing process, with input from the Data Monitoring Committee (DMC). Only the leading PI of the trial will be given access to the final cleaned data sets. Project data sets will be housed on the Project Accept Web site and/or the file transfer protocol site created for the study, and all data sets will be password-protected. Each center’s PI will have direct access to their own site’s data sets.

## Abbreviations

MNM: Nicotinamide mononucleotide
SIRT1: Sirtuin1
NAD +: Sentinel metabolite nicotinamide adenine dinucleotide
CBTI: Cognitive behavioral therapy for insomnia
NR: Nicotinamide riboside
ICSD: International classification of sleep disorders
ECG: Electrocardiograph
CONSORT: Consolidated Standards of Reporting Trials
CRF: Case report form
CTU: Clinical trials unit
DMC: Data monitoring committee
PSQI: Pittsburgh Sleep Quality Index
ESS: Epworth Sleeping Scale
ISI: Epworth Sleeping Scale
FINS: Fasting serum insulin
AST: Aspartate transaminase
ALT: Alanine transaminase
RCT: Randomized controlled trial
SD: Standard deviation
LOCF: Last observation carry forward
TSC: Trial Steering Committee
DMC: Data Monitoring Committee
